# Human adaptation to high‐altitude: A contemporary comparison of the oxygen cascade in Andean, Tibetan and Ethiopian highlanders

**DOI:** 10.1113/EP092811

**Published:** 2025-09-24

**Authors:** Ayechew A. Getu, Melissa Ilardo, Joshua C. Tremblay, Jay M. J. R. Carr, Vitalie Faoro, Philip N. Ainslie

**Affiliations:** ^1^ Centre for Heart, Lung and Vascular Health University of British Columbia Okanagan British Columbia Canada; ^2^ Department of Physiology, College of Medicine and Health Sciences University of Gondar Gondar Ethiopia; ^3^ Department of Biomedical Informatics, University of Utah School of Medicine University of Utah Salt Lake City Utah USA; ^4^ Cardiff School of Sport and Health Sciences Cardiff Metropolitan University Cardiff UK; ^5^ Institute of Mountain Emergency Medicine Eurac Research Bolzano Italy; ^6^ Research Unit in Cardio‐respiratory Physiology, Exercise & Nutrition, Faculty of Human Motor Sciences Université libre de Bruxelles Brussels Belgium

**Keywords:** acclimatization, adaptation, high altitude, oxygen cascade, native highlanders

## Abstract

Human populations native to high altitude have evolved distinct physiological adaptations to chronic hypoxia. This adaptation is evident in the O_2_ transport cascade. In this review, with brief inclusion of the related genetic adaptations, we compare the O_2_ cascade across three well‐characterized high‐altitude populations: Andeans (Aymara and Quechua), Tibetans/Sherpa and Ethiopians (Amhara and Oromo). We contrast the steps of the O_2_ cascade: (1) ventilation; (2) pulmonary O_2_ diffusion; (3) cardiac output and circulation; (4) haematological traits; and (5) tissue O_2_ utilization. Tibetans exhibit a robust hypoxic ventilatory response and efficient pulmonary diffusion capacity. They maintain preserved cardiac function with optimized muscle energetics. These adaptations are supported by enhanced tissue blood flow and greater muscle capillary density. Andeans demonstrate a blunted ventilatory response and marked remodelling of the pulmonary circulation, resulting in elevated pulmonary arterial pressure and mild but persistent right ventricular hypertrophy along with lifelong sympathetic overactivity. They also show strong haematological adaptations, with increased haemoglobin concentration. Ethiopians, particularly Amhara highlanders, show ventilatory status close to sea‐level values, with limited pulmonary and cerebral vasoreactivity to hypoxia. Although data remain limited, the Amhara highlanders exhibit higher oxygen saturation and enhanced tissue blood flow in comparison to Oromo counterparts. In conclusion, these physiological (i.e., O_2_ cascade) differences provide evidence of the diverse patterns of evolutionarily adaptive responses to the stresses of high‐altitude hypoxia. Further research comparing the O_2_ cascade across these Indigenous populations will enhance our understanding of the genetic and physiological adaptations to life at high altitude.

## Abstract (highlights)

- **What is the topic of this review?**
  This review examines contemporary research findings in the context of the oxygen cascade among the three well‐recognized high‐altitude natives: the Andeans (Aymara, Quechua), Himalayans (Tibetans, Sherpa) and Ethiopian Highlanders (Amhara, Oromo).
- **What advances does it highlight?**
  It highlights the divergent oxygen cascade in native highlanders, probably influenced by geography and evolution. Variability in study sites and limited research in Ethiopian highlanders challenges comparison and underscores the need for nuanced interpretation. For comprehensive understanding of human adaptation to high altitude, integrative approaches across the high‐altitude populations that combine genomic, physiological, anthropological and environmental studies are recommended.

## 1 INTRODUCTION

With ascent to high altitude, barometric pressure decreases along with the partial pressure of oxygen ($P_{O_{2}}$). In humans, the associated physiological strain of ascent to high altitude leads to rapid adjustments through the process of acclimatization in order to maintain tissue O_2_ supply. Acclimatization is often characterized by an increase in ventilation (${\dot{V}}_{E}$) and haemoglobin (Hb) concentration. However, these two factors alone might be inadequate to define ‘acclimatization’, because many integrative processes are involved along the O_2_ cascade that contribute to lowlanders becoming acclimatized and that contribute to the evolutionarily produced differences in high‐altitude populations. The major benefits of acclimatization, therefore, are to restore and maintain O_2_ delivery to metabolic tissues, protect against acute altitude illness, improve cognitive performance and boost submaximal exercise performance, although typically without improving the reduced peak O_2_ consumption.

Importantly, acclimatization is a short‐term physiological response and must be distinguished from the concept of evolutionary adaptation, which involves inherited characteristics present in Indigenous high‐altitude populations that have arisen as a result of natural selection (Moore, 2017). These traits serve to distinguish populations that have evolved to thrive at high altitude from even well‐acclimatized, but non‐adapted, lowlanders. Hence, the term ‘adaptation’ refers to any features of long‐term structural, functional or behavioural adjustments that increase the ability to survive and/or reproduce in a given environment. However, there is debate about whether these characteristics arise from environmental factors operating during early growth (i.e., developmental exposure to hypoxia) or owing to genetic causes. Genetic evidence supports adaptation in populations resident at high altitude for generations in South America, the Himalayas and Ethiopia (Alkorta‐Aranburu et al., 2012; Beall et al., 2010; Bigham et al., 2009; Huerta‐Sánchez et al., 2013; Scheinfeldt et al., 2012; Simonson et al., 2010; Tissot Van Patot & Gassmann, 2011; Valverde et al., 2015). Signatures of positive selection in genes related to high altitude have also been observed at lower elevations, including in Daghestani populations from the Caucasus (Laks and Kubachins, ∼2000 m; Pagani, Ayub et al., 2012) and in Papua New Guinea highlanders (2300–2700 m; André et al., 2024), although there has been insufficient physiological investigation to include these populations in this review.

When considering adaptation, populations indigenous to high‐altitude regions have developed specific, divergent adaptations over millennia living in hypoxic conditions (Beall, 2006, 2007). Although high‐altitude populations are found in various regions of the world, the best characterized are the Tibetans and Sherpa residing in the Qinghai‐Tibetan Plateau; the Andeans residing in the Andean Altiplano (including the Aymara and Quechua) of South America; and the Ethiopian high‐altitude populations residing in the Simien Plateau (the Amhara) and in the Bale Mountain region of Southern Ethiopia (Oromo). Despite the fact that only ∼ 1.4% of the population of Nepal lives above 2500 m, Tibetans are far more extensively studied than Ethiopians and Andeans, although >20% of Ethiopians, 24% of Peruvians and 54% of Bolivians live at similar elevations (Tremblay & Ainslie, 2021).

Historical records indicate that the Amhara of Ethiopia have been living at higher altitudes for ≤70 000 years, whereas the Oromos of Ethiopia are believed to have resided at high altitude for only 500 years (Simonson, 2015). Humans are thought to have occupied the Tibetan plateau for ∼30 000–40 000 years and eventually crossed a land bridge that once connected present‐day Russia and Alaska, which facilitated North and South American settlement and eventual migration to the Andes ≥11 500 years ago (Rademaker et al., 2014). There is evidence of human presence at >4000 m in the Andes dating ≥11 500 years (Rademaker et al., 2014). [Correction made on 11th October 2025, after initial online publication: The citation ‘Steffen, 2024’ has been replaced with ‘Rademaker et al., 2014.’] Early modern humans occupied the Tibetan Plateau ∼30 000–40 000 years ago, and Denisovans, an extinct archaic hominid, were present on the Tibetan Plateau 160 000 years ago (Wang et al., 2023). The unique ancestry of modern Tibetans can be traced ≥5100 years (Wang et al., 2023).

In this review, with brief inclusion of the related genetic adaptations, we discuss contemporary research findings in the context of the O_2_ cascade among the three well‐recognized high‐altitude populations. We compare the key phenotypes characterizing each step of the O_2_ cascade between Tibetans, Andeans and the Ethiopian high‐altitude populations and outline future research questions. When appropriate, we also discuss the alterations that occur in acclimatized lowlanders and compare them with native highlanders.

## 2 OVERVIEW OF OXYGEN CASCADE IN HIGH‐ALTITUDE POPULATIONS

The O_2_ cascade refers to the physiological process by which O_2_ moves from the ambient air to the mitochondria, where it is ultimately used. This process encompasses multiple steps, beginning with the convective flow of O_2_ into the alveoli, which determines alveolar $P_{O_{2}}$ ($P_{AO_{2}}$) at any given altitude. The movement of O_2_ is driven by pressure gradients between the atmosphere, lungs, blood and tissues, with the lowest O_2_ concentration occurring at the mitochondrial level. The lungs, alveolar–capillary membranes, cardiovascular system, Hb concentration and volume, tissue capillary density and mitochondrial volume are all crucial components of the O_2_ cascade. Together, they ensure effective O_2_ delivery and utilization across different altitudes. As described in the following sections, the reduction in inspired $P_{O_{2}}$ ($P_{IO_{2}}$) is met with various physiological adjustments along the oxygen transport pathway. One major consequence is a reduced pressure gradient across both the alveolar–capillary membrane and the tissue capillary–mitochondrial interface, which limits diffusive oxygen transport. In acclimatized and adapted individuals, a progressive reduction in $P_{O_{2}}$ at each stage (e.g., alveoli, arterial blood, capillaries) is associated with a less steep $P_{O_{2}}$ reduction in subsequent compartments, ultimately preserving mitochondrial oxygen availability. In other words, the diffusion gradient could be maintained through mechanisms such as hypoxic pulmonary vasoconstriction and enhanced ventilation at the lung level, while convective oxygen delivery is improved by an increase in cardiac output and other circulatory adaptations. In the following sections, our narrative review will explore each step of the O_2_ transport cascade, focusing on the three best‐known native high‐altitude populations.

## 3 STEP 1. VENTILATION

At high altitudes, where O_2_ pressure is reduced, the respiratory system compensates by increasing alveolar ventilation (${\dot{V}}_{A}$). This response, the most important facet of early acclimatization, is mediated primarily by peripheral chemoreceptors, a mechanism often referred to as the acute hypoxic ventilatory response (HVR). Typically, during acute exposure to hypoxia, ${\dot{V}}_{A}$ initially rises to a peak within the first few minutes, then decreases slightly, stabilizing at a level higher than baseline, with the extent depending on the severity of the hypoxia (Easton et al., 1986; Powell et al., 1998). This reduction in ${\dot{V}}_{A}$ is known as the hypoxic ventilatory decline (HVD) and is typically measured using protocols that involve prolonged hypoxic exposure lasting >10 min. The alveolar $P_{O_{2}}$ ($P_{AO_{2}}$), is directly influenced by ${\dot{V}}_{A}$. Additionally, ${\dot{V}}_{A}$ has an inverse relationship with the alveolar partial pressure of carbon dioxide ($P_{\mathrm{AC}O_{2}}$) (Equation 1):

(1) $${\dot{V}}_{A}=\frac{{\dot{V}}_{CO_{2}}}{P_{\mathrm{AC}O_{2}}}\times K$$

Where: ${\dot{V}}_{A}$ is alveolar ventilation, ${\dot{V}}_{CO_{2}}$ is the rate of CO_2_ production, *K* is a constant (usually 0.863), and $P_{\mathrm{AC}O_{2}}$ is the alveolar partial pressure of CO_2_.

It is important to recognize that $P_{AO_{2}}$ is directly proportional to $P_{IO_{2}}$ and inversely proportional to $P_{\mathrm{AC}O_{2}}$. However, the relationship between O_2_ consumption and carbon dioxide production is not 1:1. Rather, for every mole of O_2_ consumed, ∼0.8 moles of CO_2_ are produced, known as the respiratory exchange ratio (*R*). Thus, the decrease in $P_{\mathrm{AC}O_{2}}$ attributable to increased ${\dot{V}}_{A}$, as observed during acclimatization (Tymko et al., 2022), does not perfectly match the increase in $P_{AO_{2}}$. This is why the rise in $P_{AO_{2}}$ tends to exceed the reduction in $P_{\mathrm{AC}O_{2}}$ during hyperventilation. These relationships can be summarized in the alveolar gas equation (Equation 2):

(2) $$P_{AO_{2}}=P_{IO_{2}}-\frac{P_{\mathrm{AC}O_{2}}}{R}+F$$

Where *F* is a small correction factor, usually ∼2 mmHg.

Next, we discuss how this initial step in the O_2_ cascade varies between indigenous high‐altitude populations and lowlanders.

### 3.1 Ventilation and hypoxic ventilatory response in Tibetans/Sherpas, Andeans and Ethiopians

**FIGURE 1:**
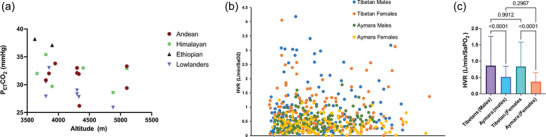
(a) Illustration of resting $P_{\mathrm{ETC}O_{2}}$ against altitude in native highlanders and acclimatized lowlanders. Each data point represents the mean value of $P_{\mathrm{ETC}O_{2}}$ and the number of studies included in the graph. Although not fully comprehensive, the general trend highlights that acclimatized lowlanders exhibit lower $P_{\mathrm{ETC}O_{2}}$ levels, indicative of heightened ${\dot{V}}_{A}$ (see Equation 1). In contrast, Ethiopian (Amhara) highlanders tend to show higher $P_{\mathrm{ETC}O_{2}}$ values, indicative of lower ${\dot{V}}_{A}$ and (b) HVR in Tibetans and Aymara (i.e., Andeans) in both males and females. Each dot represents individual data points. (c) The corresponding bar graph shows the mean and SD. HVR data are redrawn and re‐analysed from Beall et al. (1997) using WebPlotDigitizer v.4.7. Source: Andean (Beall et al., 1997; Norcliffe et al., 2005; Severinghaus et al., 1963; Slessarev et al., 2010; Stacey et al., 2023; Tymko et al., 2022); Himalayan (Beall et al., 1997; Hupperets et al., 2004; Milledge & Lahir, 1967; Tymko et al., 2022; Zhuang et al., 1993); Ethiopian (Amhara) (Gulli et al., 2007; Norcliffe et al., 2005); and acclimatized lowlanders (Hupperets et al., 2004; Milledge & Lahir, 1967; Severinghaus et al., 1963; Slessarev et al., 2010; Stacey et al., 2023; Tymko et al., 2022). Abbreviations: HVR, hypoxic ventilatory response; $P_{\mathrm{ETC}O_{2}}$, end‐tidal partial pressure of carbon dioxide.

A direct and dedicated study of ventilation and HVR in the three high‐altitude populations has not be done recently, and earlier findings were reviewed elsewhere (Beall, 2000; Brutsaert, 2007). Tibetans and Sherpas (i.e., Himalayans) seem to exhibit a heightened ventilatory response to hypoxia (Figure 1b), which is different from Andeans (Hackett et al., 1980; Zhuang et al., 1993). A previous study comparing Tibetan populations with Aymara (i.e., Andeans) natives living at altitudes of 3800–4065 m found that resting ${\dot{V}}_{A}$ was 1.5 times higher and the HVR was twice as great in Tibetans (Beall et al., 1997). Similar differences have also been observed during aerobic exercise (Beall et al., 1997). Although slightly higher altitude or lower O_2_ saturation ($S_{pO_{2}}$) in Tibetans might partly explain variations between Himalayan and Andean populations, these factors were estimated to account for only a minor portion of the observed differences (Beall et al., 1997). Interestingly, ${\dot{V}}_{A}$ in Aymara females was typically less than that of Aymara males. This is somewhat surprising, because many measurements of resting ${\dot{V}}_{A}$ at high altitude indicate that females have higher values. For example, in her early studies of $P_{\mathrm{AC}O_{2}}$ in the Colorado mountains, it was reported that females had a lower $P_{CO_{2}}$ than males at the same altitude, indicating that they had higher ${\dot{V}}_{A}$ (FitzGerald, 1914). This finding is consistent with observations that Andean females at 4300 m have a higher ${\dot{V}}_{A}$ during their luteal phase that is associated with higher concentrations of progesterone, a known respiratory stimulant (León‐Velarde et al., 2001).

A striking feature of Figure 1a is the variability of the resting HVR in Tibetans and the corresponding fact that many Tibetans have higher resting ${\dot{V}}_{A}$ (figure not shown) than Aymara (Beall et al., 1997). As shown in Figure 1b, the Aymara have a fairly uniformly low HVR, and it is higher in the Tibetans.

Early work by Severinghaus et al. (1966) in both healthy and polycythaemic Andean highlanders found that the HVR was severely blunted in highlanders with chronic mountain sickness (CMS) compared with acclimatized lowlanders (Severinghaus et al., 1966). Furthermore, the relatively high HVR in Tibetans and low HVR in Andeans might well be attributable to genetic factors (Brutsaert et al., 2005). For example, the Tibetan genome exhibits an adaptive signal within *EPAS1* (Beall et al., 2010), a gene that codes for hypoxia‐inducible factor‐2α (HIF‐2α) (Tissot Van Patot & Gassmann, 2011), and there is evidence that this transcription factor plays an important role in many potential mechanisms for adaptation, including induction of the tyrosine hydroxylase gene that increases the chemosensitivity of the carotid body (Macias et al., 2018).

Lahiri et al. (1976), however, argued that the diminished hypoxic sensitivity (i.e., blunted HVR) observed in Andean adult high‐altitude natives is determined primarily by lifelong exposure to environmental hypoxia, rather than by genetic factors. This conclusion was based on their findings that all adults studied >22 years of age showed a blunted HVR, whereas only 17.2% of teenagers aged 13–20 years demonstrated a similar response. Interestingly, ∼96% of children <12 years of age exhibited an enhanced HVR. In addition, and earlier report by Forster et al. (1971) outlined that the degree of blunting (i.e., desensitization) was influenced by the timing of exposure (i.e., blunted response to hypoxia later in adulthood if chronic exposure is begun during childhood), duration and intensity of chronic exposure, indicating that the notion that HVR might be dependent on specific genetic traits is challenged.

As previously described, a heightened ventilatory response during sojourn to high altitude is the expected and crucial feature of ventilatory acclimatization. Lowlanders acclimatized to high altitude have shown a pattern of ventilatory response to hypoxia that is different from native highlanders. For example, Slessarev et al. (2010) reported that lowlanders acclimatized to high altitude (After 10 days at 3850 m) showed lower basal ventilation and an enhanced ventilatory sensitivity to CO_2_ with similar decrease in ventilatory recruitment threshold to hypoxia in comparison to native Andeans. Bhaumik et al. (2003) reported an elevated HVR after staying at 4350 m for 1 week compared with HVR at 2100 m in both lowland males and females. Frost et al. (2024) also reported that acclimatization to high altitude resulted in increased HVR and that a decrease the ventilatory recruitment threshold contributed to augmented HVR. Interestingly, these ventilatory responses have also been investigated in the context of high‐altitude performance. Masuyama et al. (1986) found that a higher HVR was significantly associated with better performance in mountain climbers; however, this finding was not supported by others (Bernardi et al., 2006), who found that successful climbers at extreme high altitude (without oxygen) had smaller responses to hypoxia during acclimatization.

As noted, the majority of studies have reported elevations in HVR in Tibetans in comparison to Andeans (reviewed by Beall, 2000). At first glance, however, this seems to be in clear discordance with arterial blood gas data (Hoiland et al., 2019) and Figure 1a, which generally shows higher end‐tidal $P_{CO_{2}}$ ($P_{\mathrm{ETC}O_{2}}$) levels in Himalayan residents, which is more consistent with a lower ${\dot{V}}_{A}$. We interpret this difference to be explained by the nature of HVR and ${\dot{V}}_{A}$ measures. The HVR reflects the sensitivity of the carotid bodies to an acute hypoxic stimulus. In contrast, the mechanisms regulating steady‐state ventilation reflect the well‐established independent roles of the peripheral and central chemoreceptors, cardioventilatory control based on plasticity of chemosensitivity, multiple sites of hypoxic sensing, interdependence of central and peripheral chemoreceptors and upregulation of CNS neurons composing the respiratory and sympathetic regulatory pathways (reviewed by Luks et al., 2021). Such factors are likely to be regulated in a manner that explains the temporal changes in ventilation over time at high altitude, including the variability and reported ventilatory depression in some populations native to high altitude.

It is worth nothing here that a recent comprehensive meta‐analysis study observed a wide range of HVR among high‐altitude natives (Oeung et al., 2023), including Andeans who were previously found to have blunted HVR (Beall et al., 1997; Lahiri et al., 1976). However, the authors confirmed that low HVR in sea‐level residents measured at sea level was similar to that observed in Andean groups (Oeung et al., 2023). Based on their data, it seems that the previous reports of a blunted HVR might not be unique to Andeans. This meta‐analysis study and the findings highlight the high level of variation in HVR within and across individuals and populations, both in lowlanders and in native highlanders. Although controversial, the variability in HVR might have implications in determining an individual's susceptibility to hypoxia‐related pathologies, including obstructive sleep apnoea (Narkiewicz et al., 1999), development of acute mountain sickness (Moore et al., 1986) and high‐altitude pulmonary oedema (Matsuzawa et al., 1989). However, HVR status does not appear to be associated with CMS (Kryger et al., 1978; Leo´n et al., 2003).

No studies have yet characterized the ventilatory status and hypoxic ventilatory response of Ethiopian high‐altitude natives. However, higher resting $P_{\mathrm{ETC}O_{2}}$ has been reported in Ethiopian Amhara native high‐altitude residents at 3622 m compared with Peruvian (i.e., Andean) highlanders (38 ± 1 versus 29 ± 1 mmHg; Claydon et al., 2008). This implies that Amhara native highlanders do not hyperventilate at this altitude (according to Equation 1). Interestingly, relative hypoventilation was evident in Ethiopians (Amhara) 1 day after descent to 794 m, with $P_{\mathrm{ETC}O_{2}}$ levels reaching 50 ± 1 mmHg (Claydon et al., 2008). From Equation 1, considering a resting ${\dot{V}}_{CO_{2}}$ of 200 mL/min (Nunn, 1993), the ${\dot{V}}_{A}$ corresponds to 3.5 L/min at low altitude and ∼4.4 L/min at high altitude. Whether this is true for Oromo native highlanders in the Bale Mountains of Southern Ethiopia remains to be investigated.

## 4 STEP 2. PULMONARY OXYGEN DIFFUSION/GAS EXCHANGE

As fresh inspired gas is being delivered to the alveoli by the process of ${\dot{V}}_{E}$, it becomes saturated with water vapour and mixed with gas resident in the alveoli. Then, by simple diffusion, O_2_ must traverse the alveolar–capillary membrane, transfer into the blood and bind to haemoglobin in red blood cells, with the exchange of CO_2_ (Agostoni et al., 2011). Diffusion across this barrier between the alveolar spaces and the pulmonary capillaries can be conceptualized as conductance, or the ‘ease of transfer’ of gas across the membrane, and is governed by the Fick's law of diffusion (Equation 3). The law notes that diffusion of gas across a membrane is directly proportional to the area of tissue (*A*), the pressure gradient across the membrane (*P*_1_ − *P*_2_) and the diffusion constant for the particular gas (*D*) and is inversely proportional to the thickness of the membrane (*T*):

(3) $${\dot{V}}_{\mathrm{gas}}\propto \frac{A}{T}\times D\left(P_{1}-P_{2}\right)$$

Additionally, according to the Roughton–Forster equation (Equation 4), this diffusion process is influenced by two resistive components in series: the ‘membrane diffusion component’, which reflects how efficiently gas molecules move from the alveoli to the pulmonary capillary blood, and the ‘vascular diffusion component’, which represents the capacity of red blood cells and haemoglobin to take up O_2_ in the pulmonary capillaries (Hughes, 2022):

(4) $$\frac{1}{D_{L}}=\frac{1}{D_{m}}+\frac{1}{\theta V_{c}}$$

**FIGURE 2:**
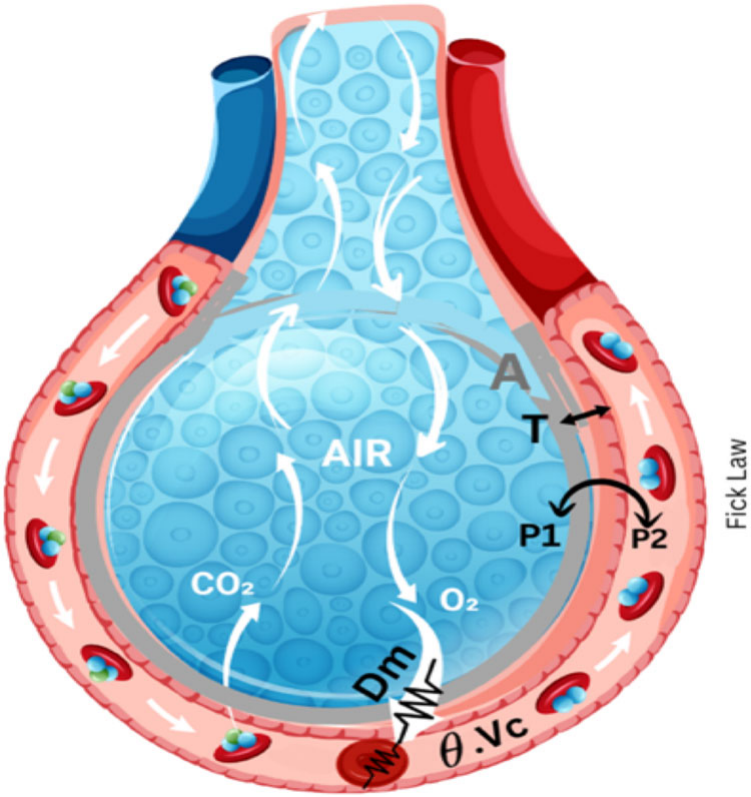
Illustrations of the mechanisms of gas diffusion across the alveolar–capillary membrane: Fick's law: Surface area of alveolar–capillary membrane (*A*); the thickness of the alveolar–capillary membrane (*T*); partial pressure of gas in alveolus (*P*_1_); partial pressure of gas in pulmonary capillaries (*P*_2_); Roughton–Forster law: diffusion capacity of the membrane (*D*_m_); representative of the resistance for gas transfer (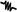); reaction rate of gas binding to haemoglobin (θ); and capillary blood volume (*V*_c_).

Where *D*_m_ is the diffusing capacity, based on molecular diffusion of the membrane separating the alveolar air from the red cell membrane; *D*_L_ is the overall diffusing capacity of the lung, *V*_c_ is the total volume (in millilitres) of blood exposed to alveolar air, θ is the number of millilitres of gas taken up by the red boold cells in 1 mL of blood per minute per 1 mmHg gradient of partial pressure of dissolved gas between plasma and the red blood cell interior. Hence, in addition to properties of the alveolar–capillary interface, diffusion will depend on the pulmonary capillary blood volume effectively participating in gas exchange (*V*_c_) and how readily haemoglobin molecules take up O_2_ (Figure 2).

Lung diffusion capacity, assessed by the diffusion capacity of the lungs for carbon monoxide (DLCO), increases with altitude and increases further with acclimatization, which leads to an increased arterial O_2_ content. It has been suggested that an increase in alveolar volume, a reduction in resistance across alveolar–capillary membrane and haemoglobin concentration contribute to enhanced lung diffusion (Agostoni et al., 2011). Early studies indicate that adult Andeans at high altitude exhibit larger lung volumes and also significantly higher DLCO (>30%) compared with acclimatized lowlanders (Greksa, 2006; Vincent et al., 1978). This adaptation is also observed in Andean children, probably owing to early alveolar proliferation and increased lung growth in response to chronic hypoxia at high altitude (Vargas ’ et al., 1982). These insights have prompted the development of recent altitude‐adjusted DLCO reference values for Latin American populations (Vázquez‐García et al., 2016).

In Sherpas, early reports noted that the diffusion capacity was higher than in the lowland climbers (Guleria et al., 1971). This has been confirmed in a larger study showing that highlanders display higher DLCO with inceases in both *D*_m_ and V_c_ in comparison to sea‐level values (Horscroft et al., 2017). Those differences could be genetically determined, although results also indicate that they might result from hypoxic stimulation of lung growth during early life (Cerny et al., 1973). It has also been found that Sherpas and Andeans maintain a substantially higher diffusion capacity, not only at rest but also during exercise, which allows them to preserve arterial O_2_ despite lower ${\dot{V}}_{E}$ compared with lowlanders (Schoene et, 1990; Wagner et al., 2002).

**FIGURE 3:**
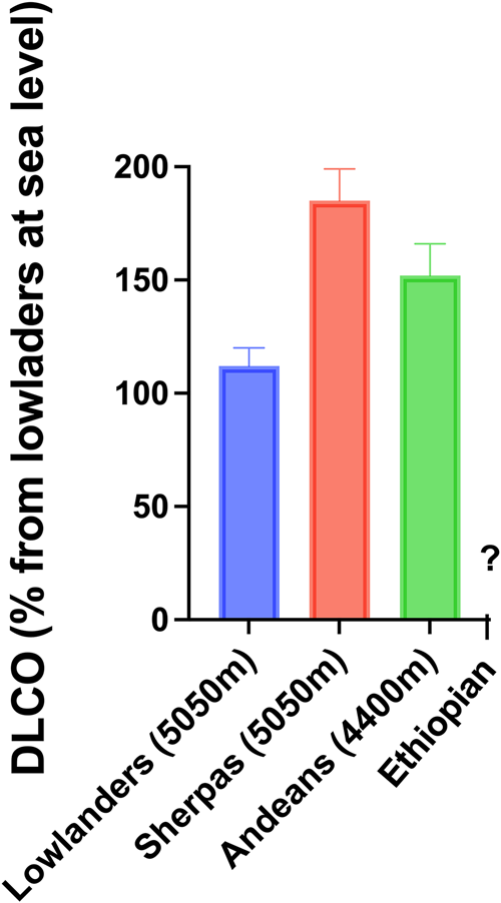
The pulmonary diffusion capacity of Sherpas, Andeans (Quechuas) and acclimatized lowlanders as measured by DLCO presented as percentage of the value for lowlanders at sea level. The figure illustrates that Sherpas and Andean (i.e., Quechuas) exhibit a greater pulmonary diffusion than acclimatized lowlanders. The bar graph shows the mean percentage changes and standard deviation. Source: de Bisschop et al. (2010); Faoro et al. (2014). Abbreviation: DLCO, diffusion capacity of the lungs for carbon monoxide.

Researchers have also investigated the contributions of the membrane and capillary components to overall diffusion capacity by incorporating concurrent nitric oxide diffusion capacity (DLNO) measurements alongside DLCO. DLNO primarily reflects membrane diffusion capacity owing to the extremely high, almost infinite, affinity of Hb for NO. Studies measuring DLNO and DLCO in high‐altitude populations from the Himalayas and Andes have consistently reported significantly higher lung diffusing capacities for both gases in comparison to lowlanders acclimatized to high altitude (de Bisschop et al., 2010; Faoro et al., 2014; Groepenhoff et al., 2012). Notably, these elevations persist even after adjusting for alveolar volume, reflecting a superior gas exchange capacity per surface unit in high‐altitude natives, probably related to structural adaptations. Nevertheless, when corrected for alveolar volume and Hb, DLCO levels reached 153% of sea‐level reference values in Andeans and 168% in Sherpas, and DLNO reached 114% of predicted values in Andeans and 130% in Sherpas (de Bisschop et al., 2010; Faoro et al., 2014). These observations suggest an enhanced diffusion capacity of Sherpas when compared with Andeans (Figure 3).

To our knowledge, there has been no previous research examining pulmonary gas exchange or diffusion capacity, either at rest or during exercise, in the Ethiopian high‐altitude populations. Investigation is warranted in these high‐altitude groups.

## 5 STEP 3. CARDIAC OUTPUT AND CIRCULATION

The cardiovascular system is an essential component of the O_2_ delivery process, because it regulates both the uptake of O_2_ from the lung and its transport to distant metabolic tissues. The heart responds to hypoxia through a range of adaptive responses, including functional changes, metabolic remodelling and structural modifications (Essop, 2007; Williams et al., 2022). Exposure to acute hypoxia causes the heart to compensate by elevating cardiac output with an increased rate of contraction (tachycardia) while stroke volume remains unchanged (Naeije, 2010). This immediate response is largely driven by heightened sympathetic activation and chemoreflex stimulation. However, after acclimatization, cardiac output gradually decreases, owing to reductions in stroke volume while the heart rate remains elevated (Klausen, 1966). This reflects a multifactorial adjustment involving baroreflex modulation, reduced plasma volume, altered autonomic balance and enhanced peripheral O_2_ extraction. These time‐dependent adjustments reflect a shift from an acute compensatory strategy to a more energy‐efficient chronic adaptation. Cardiac adaptations to acute, prolonged and lifelong high‐altitude hypoxia have been reviewed recently (Stembridge et al., 2015; Williams et al., 2022). Here, we briefly examine how cardiac output and peripheral blood flow differ between Andean, Himalayan and Ethiopian high‐altitude natives.

One major distinction in the cardiovascular system between high‐altitude native populations is the varying degree of pulmonary vasoconstrictive response to hypoxic stimuli, which influences pulmonary arterial pressure and right ventricular afterload. For example, marked remodelling of the pulmonary circulation is evident in Andean high‐altitude natives and is associated with elevated arterial pulmonary pressure (Groepenhoff et al., 2012; Penaloza & Arias‐Stella, 2007). At altitudes between 3600 and 4350 m, healthy Andeans typically exhibit mean pulmonary arterial pressures of ∼20–23 mmHg, which have been described as similar, slightly lower or slightly higher than acclimatized lowlanders, depending on the characteristics of the control group and the altitude at which measurements were taken (Antezana et al., 1982; Groepenhoff et al., 2012; Huez et al., 2009; Patrician et al., 2022; Sakai et al., 2010). Higher pulmonary arterial pressures are typically observed in patients with CMS, along with lower $S_{pO_{2}}$ (Penaloza & Arias‐Stella, 2007), and both can be exacerbated during exercise (Groepenhoff et al., 2012; Lorenza et al., 2013).

In terms of cardiac functional adaptation to high altitude, it appears relatively similar between acclimatized lowlanders and Andean highlanders. However, lifelong hypoxic exposure might lead to subtle differences, including minor diastolic adaptation and slightly reduced right ventricular systolic performance (Huez et al., 2009). These differences might arise from variations in sympathetic nervous system tone, chronically reduced preload and, possibly, direct myocardial effects of sustained hypoxaemia (Huez et al., 2009). Concurrently, their resting cardiac output is slightly lower than that of acclimatized lowlanders, primarily owing to a reduced stroke volume (Huez et al., 2009). Notably, in Andean highlanders, cardiac output shows little variation between altitudes of 3500 and 5000 m, suggesting a stable cardiovascular adaptation to chronic hypoxia (Doutreleau et al., 2022). However, Andean highlanders with excessive erythrocytosis might exhibit a different cardiovascular status. Indeed, those with excessive erythrocytosis show higher arterial O_2_ levels and lower cardiac output, whereas those without erythrocytosis have slightly increased cardiac output to maintain similar O_2_ transport (Anza‐Ramírez et al., 2023). A genetic signature of evolution in the Andes might be related to this cardiovascular resilience. For example, genetic variants under selection in Andean populations showed overlap with the nitric oxide pathway and cardiovascular system (Bigham et al., 2010, 2013; Crawford et al., 2017). Genes under selection in Andean populations include *NOS2* and *NOS2A*, both of which encode nitric oxide synthase, and endothelin receptor type A (*EDNRA*), which plays a role in vascular control (Bigham et al., 2010, 2013; Crawford et al., 2017). Interestingly, selection on cardiovascular function in the Andean Altiplano can be dated back >7000 years, with ancient DNA supporting evidence of positive selection on *DST*, a gene involved in cardiac function (Lindo et al., 2018). In a study comparing patients with CMS and acclimatized lowlanders, Lorenza et al. (2013) reported similar left ventricular systolic and diastolic function at rest and during exercise. However, they observed lower resting right ventricular function in CMS patients but with a maintained right ventricular contractile reserve during exercise. This suggests that the resting differences reflect physiological adaptations to chronic hypoxia rather than impaired ventricular function (Lorenza et al., 2013).

**FIGURE 4:**
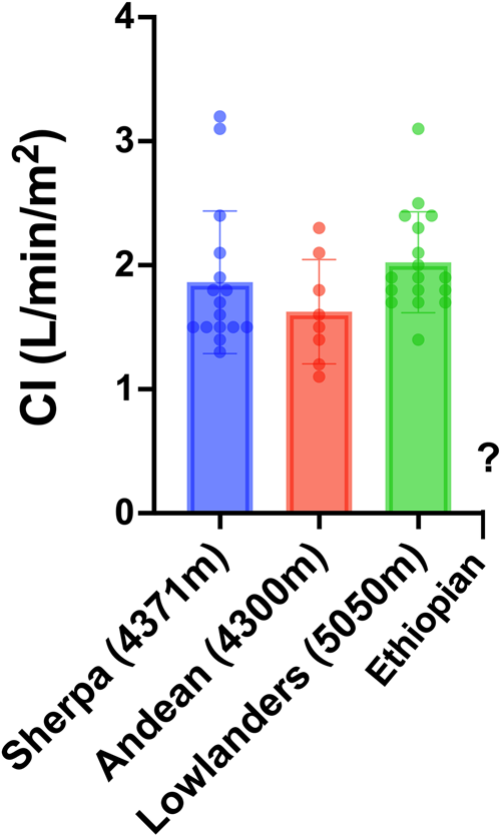
Resting cardiac index calculated as cardiac output divided by body surface area in lowlanders acclimatized to indicated high altitude versus native healthy highlanders.Cardiac index Values were calculated using raw data from Stembridge et al. (2019). Abbreviation: CI, cardiac index.

In contrast to Andeans with and without CMS, Himalayan Sherpas have long been thought to experience minimal pulmonary vasoreactivity, with a milder increase in pulmonary arterial pressure. Mean pulmonary artery pressure in Sherpas typically ranges from ∼15 to 22 mmHg, with some interindividual variability and differences across studies related to environmental and altitude conditions (Chen et al., 2022; Groves et al., 1993; Stembridge et al., 2014). Interestingly, a comparative study of Tibetans versus Han Chinese both living at sea level showed that Tibetans exhibited a blunted pulmonary vascular response to acute or sustained hypoxia, suggesting an inherent adaptation to low‐oxygen environments (Petousi et al., 2014). This supports the notion that Tibetan highlanders possess genetically determined mechanisms that might protect against hypoxia‐induced pulmonary hypertension. During exercise, Sherpas demonstrate a mild increase in pulmonary arterial pressure, indicating a large pulmonary vascular reserve (Faoro et al., 2014; Groves et al., 1993). This blunted hypoxic pulmonary vasoconstriction response is considered a significant advantage of natural selection, because it helps to maintain a lower right ventricular afterload, reducing cardiac strain at high altitude, even at exercise. Notably, when pulmonary vascular resistance is adjusted for haematocrit, the observed differences between Sherpas and Andeans are reduced, suggesting that lower blood viscosity provides an advantage by helping to limit right ventricular afterload (Groves et al., 1993). Sherpas also demonstrate a smaller stroke volume and cardiac output in comparison to lowlanders at high altitude (Stembridge et al., 2015). Nonetheless, Tibetans and Sherpas have a higher maximal heart rate at high altitude (Stembridge et al., 2015), which is likely to compensate for a reduced stroke volume to sustain high cardiac output levels at exercise. These adaptations might help to preserve cardiac reserve, ensuring efficient O_2_ transport during exercise at high altitude. Hypoxia markedly decreases maximal cardiac output, thereby affecting maximal aerobic exercise capacity (Naeije, 2010). Limited pulmonary vascular reserve might contribute to the altered maximal cardiac output in hypoxia. Several studies using pharmacological agents to decrease pulmonary vascular resistance in hypoxic conditions have shown partial restoration of maximal cardiac output with variable positive effects on aerobic exercise capacity (Hossein et al., 2004; Naeije et al., 2010). Figure 4 shows that, although the altitude difference makes comparision difficult, lowlanders acclimatized to high altitude exhibited a greater cardiac index (CI), while Andeans showed the lowest (Stembridge et al., 2019). Taken together, the evidence indicates that the alteration in maximal exercise capacity in hypoxia is explained primarily by limitations in convective oxygen transport and its matching with diffusional oxygen transport, although it might be influenced by the pulmonary vascular resistance (Naeije, 2010; Wagner, 1992).

The presence of potential cardiac adaptation in Ethiopian highlanders is not as well studied; however, Hoit et al. (2011) reported a similar Doppler estimation of pulmonary blood flow/stroke volume among Ethiopian highlanders (3700 m) and lowlanders (1200 m). Their findings revealed a unique pattern of increased pulmonary blood flow and pressure but lower pulmonary vascular resistance, suggesting distinct cardiovascular adaptations to chronic hypoxia (Hoit et al., 2011). Their data also indicate that there is no significant difference in heart rate at rest between Ethiopian highlanders and lowlanders. In summary, high‐altitude natives exhibit distinct pulmonary haemodynamics and cardiac features, reflecting functional adaptations to chronic hypoxic stress throughout their lifespan.

Vascular function in high‐altitude populations has been reviewed recently (Tymko et al., 2019). In relationship to peripheral blood flow, it has been reported that Tibetans have more than double the forearm blood flow measured using strain‐gauge plethysmography compared with low‐altitude residents, resulting in greater O_2_ delivery to tissues (Erzurum et al., 2007). This was also the case during forearm exercise, in which Tibetans had much greater flow increase than sea‐level individuals (Erzurum et al., 2007). Additionally, compared with sea‐level control subjects, Tibetans exhibited a >10‐fold increase in circulating concentrations of bioactive nitric oxide (NO) products, including plasma and red blood cell nitrate and plasma nitrite (Erzurum et al., 2007). This elevation in NO might facilitate normal O_2_ delivery in hypoxia through enhanced blood flow (Allen et al., 2009). It should be noted, however, that more recent studies using ultrasonography have not observed elevations in peripheral blood flow in Sherpa (when compared with lowlanders), at sea level or at similar high altitude (Bruno et al., 2014; Tremblay et al., 2018). A greater vascular reactivity (e.g., reactive hyperaemia) has been identified in Himalayan highlanders compared with lowlanders at high ‐altitude (Schneider et al., 2001), suggesting heightened vascular reserve (Bruno et al., 2014). Differences in the measurement of blood flow, differing elevation and groups studied might explain these divergent findings. In Andean populations, O_2_ delivery during small muscle mass exercise is preserved despite higher blood viscosities, potentially via structural (e.g., larger arteries to normalize shear stress) and functional alterations that maintain exercise capacity even with marked polycythaemia (Tremblay et al., 2019; Hansen et al., 2021). Invasive studies comparing Aymara populations (compared with Danish lowland participants) at high altitude indicate similar resting and submaximal leg blood flows during exercise (Lundby et al., 2006; Rådegran, 2008). In contrast, however, maximal leg blood flow during exercise was lower in Aymara compared with acclimatized Danish lowlanders (Lundby et al., 2006; Rådegran, 2008).

In a mechanistic study that used intra‐arterial infusion of acetylcholine and sodium nitroprusside (endothelium‐independent dilatation) before and after local α‐ plus β‐adrenergic receptor blockade (phentolamine and propranolol), chronic severe hypoxia exposure was shown to result in endothelial dysfunction in Andean highlanders, partly through adrenergic vasoconstrictor signalling (Tymko et al., 2020). The vascular dysfunction was particularly pronounced in Andeans with CMS. Recently, Savina et al. (2024) also demonstrated that skin microvascular and macrovascular function is diminished with altitude exposure in high‐altitude natives, with further impaired with CMS (Savina et al., 2024). This latter impairment was accompanied by increased systemic oxidative stress and inflammation. It appears that native Andean highlanders, particularly those affected by CMS, exhibit reduced vascular reactivity.

With regard to Ethiopian high‐altitude populations, neither peripheral blood flow at rest nor vascular reactivity has been measured. However, the Amhara high‐altitude natives (but not the Oromos) showed elevated urinary levels of nitrate and cyclic guanosine monophosphate (Cheong et al., 2017), which causes the relaxation of the vasculature (Archer et al., 1994). This suggests that the Amhara native highlanders, in particular, might have some vascular adaptive responses to chronic hypoxia.

As described elsewhere in this review, high‐altitude hypoxia is a significant challenge for every system in the body including the autonomic function. Enhanced activity of the sympathetic nervous system (i.e., sympathoexcitation) now seems a universal feature that occurs at both initial arrival at high altitude and during acclimatization, and in native highlanders, and has been implicated to regulate vasomotor tone and peripheral blood flow (Fisher et al., 2018; Simpson et al., 2019, 2021). In this regard, Sherpas appear to have lower sympathetic vasomotor outflow in comparison to both acclimatized lowlanders (Simpson et al., 2019) and healthy Andean natives (Lundby et al., 2018; Tymko et al., 2020). Andean highlanders appear to have adapted to maintain elevated sympathetic activity throughout life at high altitude, whereas Himalayan Sherpas exhibit an adaptation characterized by lower sympathetic vasomotor outflow (Simpson et al., 2021). Whether this autonomic adaptation is also characteristic of Ethiopian highlanders remains unexplored and warrants investigation. Collectively harmonized methods to assess regional blood flow distribution at rest and in response to exercise, in addition to other commonly encountered stressors, such as heat/cold, are required to understand whether peripheral blood flow (and, more importantly, vascular reserve) differs across high‐altitude populations.

## 6 STEP 4. HAEMOGLOBIN CONCENTRATION AND OXYGEN TRANSPORT IN THE BLOOD

A reduction in $P_{O_{2}}$ at high altitude, via key haematological factors, is slowly compensated by increasing the concentration of Hb (Mairbäurl & Weber, 2012). Haemoglobin concentration is one of the differential manifestations of adaptations between high‐altitude natives. Although considerable variation exists in the concentration of Hb among native highlanders (Beall, 2006), largely attributable to sample characteristics (Beall et al., 1990; Frisancho, 1988; Garruto, 1983) (e.g., urban vs. rural, occupation), the traditional view is that Tibetans demonstrate a Hb concentration that is lower than Andeans (Beall, 2000, 2007); the lower concentration is associated with greater reproductive success (Jeong et al., 2018) and exercise capacity (Simonson, 2015). Remarkably, the Himalayan populations have long been noted for their fitness and low haemoglobin at high altitude (Wu, 2006). In recent years, population‐based studies have leveraged whole‐genome sequencing data to identify positively selected variants potentially underlying these observations (Beall et al., 2010; Bigham et al., 2010; Simonson et al., 2010; Yi et al., 2010). Selection scans implicated adaptive evolution of variants in genes involved in the HIF O_2_‐sensing pathway, including *EPAS1* (endothelial PAS domain protein 1), which encodes the HIF complex subunit *HIF*‐2*α* and *EGLN1* (egl‐9 family hypoxia inducible factor 1), which, in turn, encodes *PHD2* (prolyl hydroxylase domain‐containing protein 2), which targets *HIF*‐2*α* for degradation in conditions of normoxia (Beall et al., 2010; Bigham et al., 2010; Simonson et al., 2010; Yi et al., 2010). Naturally selected variants in both genes have been associated with the decreased haemoglobin at altitude that is characteristic of Himalayan Indigenous populations (Beall et al., 2010; Jeong et al., 2014, 2018; Lorenzo et al., 2014; Peng et al., 2011; Simonson et al., 2010; Yang et al., 2017).

Like Tibetans, Andean populations have also experienced selection on the gene *EGLN1* and, more recently, *EPAS1* (Bigham et al., 2010; Lawrence et al., 2024). Interestingly, Yasukochi et al. (2020) reported the presence of significant correlation between the Hb concentration and *EGLN1* variant, suggesting that there is a strong link between haematological adaptation and genetic variant in Andean highlanders. Furthermore, Lawrence et al. (2024) identified lower haematocrit, higher $S_{pO_{2}}$ and no cases of excessive erythrocytosis in Andean males with a positively selected EPAS1 missense variant, rs570553380 (A>G, p.[His194Arg]) (Lawrence et al., 2024). Therefore, selection on the same genes, although by distinct means, seems to occur and to be associated with lower [Hb] or haematocrit in Tibetan and Andean highlanders.

**FIGURE 5:**
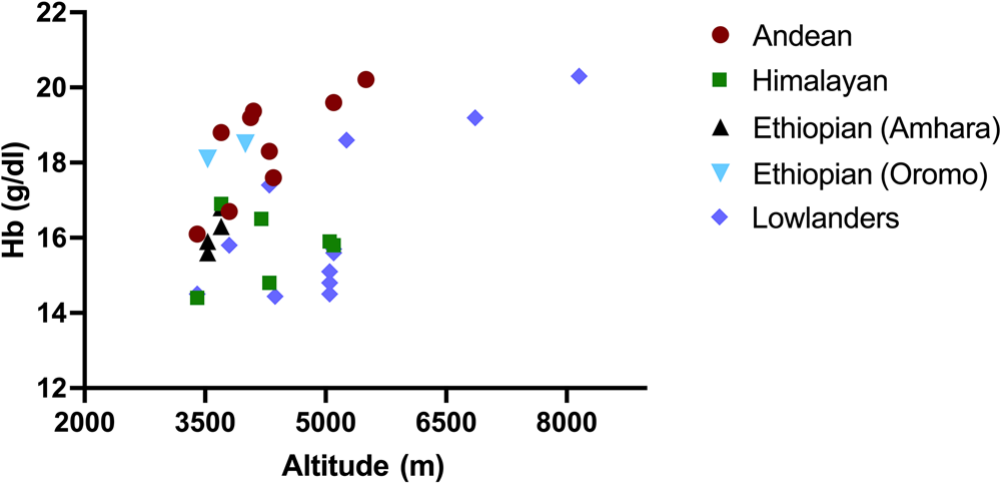
The average Hb concentration in healthy high‐altitude male natives and lowlanders acclimatized to high altitude. Each dot represents the average Hb concentration obtained from studies done at the indicated altitudes. Sources: Andean (Beall et al., 1990, 1998; Faoro et al., 2014; León‐Velarde et al., 2000; Tufts et al., 1985; Tymko et al., 2022); Himalayan (Beall et al., 1990; Erzurum et al., 2007; Faoro et al., 2014; Moore et al., 2002; Ruggiero & McNeil, 2023; Samaja & Winslow, 1979; Simonson et al., 2010; Tymko et al., 2022); Ethiopian (Amhara and Oromo) (Alkorta‐Aranburu et al., 2012; Beall, 2006; Beall et al., 2002; Hoit et al., 2011; Scheinfeldt et al., 2012); and lowlanders (Alkorta‐Aranburu et al., 2012; Beall, 2006; Beall et al., 2002; Calbet et al., 2003; Hoiland et al., 2019; Hoit et al., 2011; Johnson et al., 2025; Pugh, 1964; Scheinfeldt et al., 2012; Tymko et al., 2022; Vizcardo‐Galindo et al., 2023). Abbreviation: Hb, haemoglobin.

The Ethiopian Amhara (but not Oromos) have also been reported to have relatively low Hb concentration compared with Andeans (Beall et al., 2002; Cheong et al., 2017). Figure 5 shows population haemoglobin concentrations, demonstrating the higher concentrations seen in the Andeans and relatively low concentrations in the Tibetans and Ethiopians. In lowlanders moving to high altitude, the Hb concentration increased but varied with altitude and the duration of their stay (Rasmussen et al., 2013). It can take weeks to months to reach a new steady state that helps to restore blood O_2_ levels (Pugh, 1964).

### 6.1 Blood compartment volume and haemorheology: Candidate traits for high‐altitude adaptation?

It has been widely assumed that the lower Hb concentration in Tibetans is achieved via the absence of a significant erythropoietic response to hypoxaemia and is beneficial owing to the resultant lower (although infrequently measured) blood viscosity (Simonson et al., 2010). However, this disregards the equally important contribution of plasma volume in the regulation of haematocrit (Donnelly, 2003). A larger plasma volume would decrease haematocrit and could decrease heart rate by mediating a larger stroke volume at given cardiac output, but the importance of these volumetric measures has only recently been explored. It has been found, for example, that Hb mass is highest in Andeans, but also elevated in Sherpas compared with lowlanders at the same high altitude (Stembridge et al., 2019). Sherpas, however, demonstrated a larger plasma volume than Andeans and lowlanders, resulting in a comparable total blood volume at a lower Hb concentration. Recent findings have reported no differences in blood volume or plasma volume (including ascent‐related plasma volume contraction) between Sherpas and lowlanders at sea level or high altitude, including a similar increase in Hb mass and plasma volume contraction during exposure to 5400 m (Roche et al., 2024). Ideally, future work will assess blood volume compartments in Himalayan highlanders in residents at their resident altitude (e.g., 2500–4500 m), rather than during ascent to higher altitudes (>5000 m). Nevertheless, Sherpas and lowlanders demonstrate lower blood viscosity at high altitude than Andeans (Tremblay et al., 2019; Tymko et al., 2021). This reduced viscosity enables Himalayans to maximize total O_2_‐carrying capacity of the blood without the detrimental effect of a high viscosity on microcirculatory blood flow; the importance of haemorheological factors for blood flow at high altitude (via increases in resistance and shear stress‐mediated vasodilatation) has received greater attention recently (Cabrales et al., 2020; Steele et al., 2024). The relationship between Hb (and haematocrit) and blood viscosity is exponential (Tremblay et al., 2019; Wells & Merrill, 1962). Furthermore, blood viscosity is altered by temperature, becoming more viscous at cooler temperatures and less viscous at warmer temperatures (Rand et al., 1964). Therefore, when comparing across populations, it is important to control the temperature of blood viscosity measures. Whether this mechanism of blood volume and rheology‐mediated adaptation is present in Ethiopian native highlanders requires investigation (Roche et al., 2024). Therefore, future studies should prioritize, when possible, volumetric (plasma volume, red cell volume, blood volume and haemoglobin mass) and viscosity measures to understand the implications of Hb values.

The increase in haematocrit and blood viscosity also affects the resistance to blood flow in the pulmonary circulation (Vanderpool & Naeije, 2018). Because pulmonary vascular resistance increases exponentially with rising haematocrit, this influence is more pronounced at higher haematocrit levels, as seen in CMS patients. In fact, a recent study showed that a rapid haemodilution in Andean natives with excessive erythrocytosis led to an acute drop in pulmonary vascular resistance (Stembridge et al., 2021). Furthermore, when pulmonary vascular resistance is adjusted for haematocrit, the higher values seen in Andeans compared with Sherpas are reduced (Faoro et al., 2014). However, the extent to which haematocrit influences the right ventricular function, maximal cardiac output and aerobic capacity remains to be elucidated.

### 6.2 Oxygen saturation in high‐altitude natives

**FIGURE 6:**
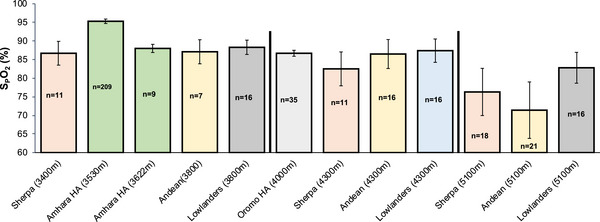
Average oxygen saturation plotted against altitude for Tibetans, Andeans, Ethiopians and acclimatized lowlanders (Europeans). Each bar represents the mean $S_{aO_{2}}$/$S_{pO_{2}}$ values, with error bars indicating standard deviations. Sample sizes (*n*) for each study are displayed. Although variability exists among altitudes and locations studied, the Amhara (3520 m) highlanders of Ethiopia showed the highest saturation ($S_{pO_{2}}$) and the Andeans (5100 m) showed the lowest saturation ($S_{aO_{2}}$). Source: (Alkorta‐Aranburu et al., 2012; Tymko et al., 2022). Abbreviations: $S_{aO_{2}}$, arterial O_2_ saturation (measured directly from arterial blood gas analysis); $S_{pO_{2}}$, peripheral O_2_ saturation (estimated non‐invasively using pulse oximeter).

Acute exposure to high altitude (>3000 m) causes a rapid and significant drop in O_2_ saturation, which is indicative of the lower partial pressure of O_2_ (Beall, 2006). Measurements of $S_{aO_{2}}$ at high altitude provide a direct assessment of the severity of hypoxaemia and the efficiency of pulmonary gas exchange under reduced ambient oxygen pressure. Figure 6 shows O_2_ saturation at various altitudes in Tibetans and Andeans in comparison to acclimatized lowlanders ($S_{aO_{2}}$ measured via arterial blood gases) and in Ethiopian Native highlanders estimated by pulse oximetry ($S_{pO_{2}}$). Based on the higher ${\dot{V}}_{E}$ and HVR, we would expect a higher $S_{aO_{2}}$ in Tibetans than in Andeans. This seems to be true above 5000 m, but some samples between 3000 and 5000 m are not in agreement with the above report (see Figure 6). In Ethiopians, however, the O_2_ saturation was estimated by $S_{pO_{2}}$, which might not be as accurate as direct measurements (Luks & Swenson, 2011). As seen in Figure 6, high‐altitude natives vary widely in O_2_ saturation despite relatively uniform ambient hypoxic stress. This indicates that genetic factors might account for much of the observed variation in oxygen saturation among these high‐altitude populations. For example, it has been proposed that genetic factors might underlie differences in $S_{aO_{2}}$ observed among Tibetan mothers, with higher $S_{aO_{2}}$ being associated with reduced infant mortality in this population (Beall et al., 2004). However, the specific genetic variants underlying these differences have yet to be identified clearly. Additionally, $S_{aO_{2}}$ is higher in Tibetan relative to Han Chinese or Andean infants at comparable altitudes, and such differences are hypothesized to underlie susceptibility to high‐altitude pulmonary hypertension and CMS later in life in the latter populations (Niermeyer et al., 2015).

**FIGURE 7:**
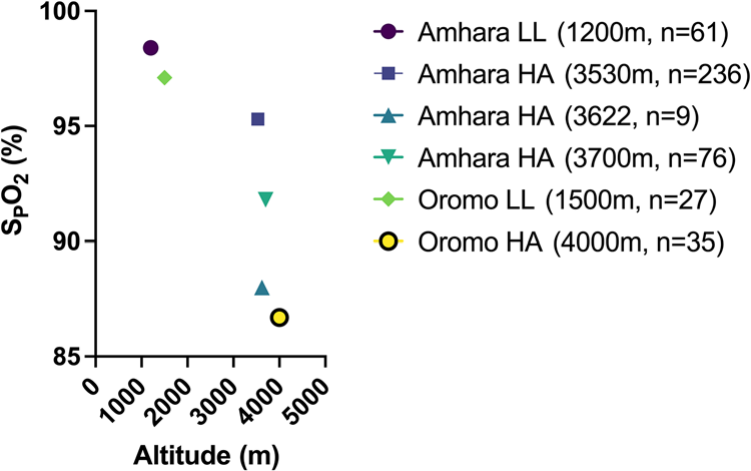
Average $S_{pO_{2}}$ in Amhara and Oromo high‐altitude natives and lowlanders. Each dot represents the average percentage saturation obtained from studies done at the indicated altitudes. The Amhara high‐altitude natives showed a greater $S_{pO_{2}}$ compared with Oromo counterparts, although the altitude level was relatively higher for the Oromo (4000 m). Sources: Alkorta‐Aranburu et al. (2012); Beall et al. (2002); Claydon et al. (2008); Hoit et al. (2011). Abbreviations: HA, highlanders; LL, lowlanders; $S_{pO_{2}}$, peripheral O_2_ saturation.

Although the $S_{pO_{2}}$ level reported in Ethiopian high‐altitude natives is scattered, the overall range at high altitude was at first reported close to sea‐level values (average $S_{pO_{2}}$ of 95%; Figure 7; Beall et al., 2002). In a separate study focusing on Amhara native highlanders (3700 m), the average $S_{pO_{2}}$ was lower than in Amhara lowlanders (1200 m; Figure 7; Hoit et al., 2011). In another study (Claydon et al., 2008), Amhara high‐altitude natives at 3622 m were shown to have an average $S_{pO_{2}}$ of 88%. In a separate study conducted at high altitudes (3700–4000 m) (Alkorta‐Aranburu et al., 2012), both the Oromo (4000 m) and Amhara (3700 m) native highlanders had lower average $S_{pO_{2}}$ of 87% and 92%, respectively, compared with sea‐level values ($S_{pO_{2}}$ = 97%); however, the Oromo had significantly lower $S_{pO_{2}}$ than the Amhara.

In summary, based on the above studies, probably owing to intrinsic variability in ventilatory control and diffusion capacity, there is a considerable variation in $S_{pO_{2}}$ among highland groups (even within the same geographical region). Nevertheless, although there is also considerable variation in altitude residence, it appears that $S_{pO_{2}}$ is highest among Amhara high‐altitude natives and is relatively lower in Andeans. Arterial blood gases with estimation of the PO_2_ at which 50% of hemoglobin is saturate  *P*_50_ values are now needed to confirm and extend these findings.

### 6.3 *P*_50_ and haemoglobin–oxygen affinity in high‐altitude natives

Increased haemoglobin–O_2_ binding affinity has typically been regarded as beneficial at altitude by enhancing the rate of equilibration across the alveolar–capillary barrier. Although reports of haemoglobin–O_2_ binding affinity differ widely across studies, recent data indicate greater O_2_ binding affinity (i.e., a decreased *P*_50_) in Tibetans and Han Chinese individuals residing for ≥2 years at 4200 m (Simonson et al., 2014) and Andean residents at high altitude (Balaban et al., 2013) compared with lowlanders at sea level. A reduction in *P*_50_ allows maintenance of higher $S_{aO_{2}}$ at high altitude but is also likely to require some adaptation at the tissue level to account for the reduction in O_2_ offloading.

**FIGURE 8:**
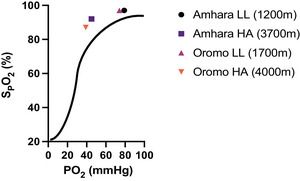
Increased affinity of haemoglobin for O_2_ in high‐altitude populations, relative to the standard O_2_ dissociation curve. Amhara and Oromo both show an increased affinity for O_2_ at high altitude. Modified from Cheong et al. (2017). Abbreviations: HA, highlanders; LL, lowlanders; PO_2_, Partial pressure of O_2_; $S_{pO_{2}}$, peripheral O_2_ saturation.

In the only study of Hb–O_2_ affinity in East‐African high‐altitude natives at 3700 m (Amhara) and 4000 m (Oromo), a higher O_2_ affinity was reported relative to the predicted standard O_2_ dissociation curve (Cheong et al., 2017). The average measured $S_{pO_{2}}$ values of high‐altitude Amhara and Oromo (Cheong et al., 2017) lie on the left side of the standard O_2_ dissociation curve (Figure 8). This suggests an increased affinity of Hb for O_2_ at high altitudes. Considering the differences in altitude at which measurements are taken and the limited available data, the greater Hb affinity reported for O_2_ among Amhara compared with Oromo should be interpreted with caution, and further measurements are needed to confirm these findings.

## 7 STEP 5. CAPILLARY AND MITOCHONDRIAL VOLUME DENSITY AND OXYGEN UTILIZATION

The microcirculation (i.e., blood vessels <100 µm in diameter) regulates localized blood flow to match tissue O_2_ demand. As the final step in the convective portion of the O_2_ cascade, alterations in the structure or function of the microvasculature might disrupt the balance between O_2_ supply and demand at a cellular level. A capillary network that is dense facilitates greater O_2_ delivery to tissues. In this regard, the microcirculation might be crucial in adaptation to chronic hypoxia. It has been demonstrated that Sherpas have a higher sublingual microcirculatory blood flow and greater capillary density at high altitude than lowlanders (Gilbert‐Kawai et al., 2017). In another study, Sherpa skeletal muscle was reported to have a greater capillary density, that is, a higher number of capillaries per cross‐sectional area, compared with sedentary lowlanders (Kayser et al., 1991).

A key component of adaptation of the tissues to hypoxia is the optimization of energy metabolism at the mitochondrial level. For oxidative tissues, in the face of hypoxia, it is crucial to adjust metabolically and reduce the burden of oxidative stress as hypoxia induces oxidative stress (Guzy & Schumacker, 2006). This can involve changes in gene expression that alter the functioning of mitochondria and their volume (Mazure & Pouysségur, 2010). For example, with prolonged exposure to extreme high altitude, acclimatized lowlanders had a loss of muscle mitochondrial volume density (Levett et al., 2012). Sherpas also have lower mitochondrial volume than lowlanders (Horscroft et al., 2017). However, compared with lowlanders, Sherpas demonstrated a lower capacity for fatty acid oxidation in skeletal muscle biopsies, along with enhanced efficiency of O_2_ utilization (Horscroft et al., 2017), suggesting a maximizing of the ratio of O_2_ consumption to mitochondrial volume. Moreover, markers of oxidative stress are elevated in the muscle of lowlanders when compared with Sherpas (Gelfi et al., 2004; Horscroft et al., 2017). With progressive altitude, there is a gradual reduction in phosphocreatine and ATP levels in lowlanders that are maintained in the Sherpas (Horscroft et al., 2017). It seems apparent that the optimized muscle energetics displayed by the Sherpas is possibly the result of adaptation at the metabolic level.

In the limited reports of muscle biopsies in Andean high‐altitude natives (Desplanches et al., 1996; Rosser & Hochachka, 1993), it appears that muscle capillary density is reduced along with muscle tissue oxidative capacity compared with acclimatized lowlanders. The numbers of capillaries supplying similar‐sized muscle fibres is reduced in proportion to the reduction of mitochondria within these fibres. Chronic hypoxia shifts the trend of muscle metabolism from oxidative to glycolytic metabolism. The density of tissue capillaries and mitochondria in Ethiopian highlanders is not known, and investigation is needed to understand the role of muscle energetics in development of hypoxia tolerance.

## 8 CONCLUSION AND FUTURE DIRECTIONS

The framework for investigating human adaptation to high altitude remains the multiple population–single stressor model; in other words, comparing how separate populations have adapted to life at high altitude. High‐altitude residents of the South American Andes, the Tibetan plateau and the Ethiopian highlands whose ancestors lived at altitude for generations have developed distinct traits, with distinct trade‐offs, while living in their respective hypoxic environments. However, it is important to note that a single stressor can mask important environmental, cultural and socioeconomic differences aside from hypoxia that can impact physiology (Tremblay, 2024). Nevertheless, while acknowledging this important point as a future research direction, the varied strategies used by Indigenous highland populations (Himalayan, Andean and Ethiopian) offer important insights into how humans have adapted to high‐altitude hypoxia. These groups display different physiological adaptations, perhaps influenced by geographical and evolutionary contexts, although they live under relatively similar hypoxic stress. It is important to note that findings about O_2_ cascades, even among the well‐studied Indigenous populations of the Andes and the Himalayas, are frequently inconsistent and contradictory. Moreover, it is noteworthy that even more closely related ethnic groups with slight differences in altitude (Amhara and Oromo), which vary in their duration of residence at high altitude, also show clear differences in some of the physiological traits (e.g., $S_{pO_{2}}$ and Hb). Thus, the substantial genetic variability between these populations might underpin interpopulation differences in the O_2_ delivery cascade. Population‐specific genetic variants and their associated physiology also provide evidence of the diverse patterns of evolutionarily adaptive responses to the stresses of high‐altitude hypoxia. Further research comparing the O_2_ cascade across these Indigenous populations will enhance our understanding of the genetic and physiological adaptations to life at high altitude. Finally, to improve on the aforementioned single stressor model, we encourage interdisciplinary research approaches that integrate genomics, physiological, anthropological and environmental sciences to develop a more comprehensive understanding of how the human body adapts to the persistent challenge of high altitude across generations.

## AUTHOR CONTRIBUTIONS

Ayechew Getu and Philip N. Ainslie conceived and designed the review; Ayechew Getu, Philip N. Ainslie and Joshua C. Tremblay drafted the review; Ayechew Getu and Vitalie Faoro, prepared the figures; Ayechew Getu, Melissa Ilardo, Joshua C. Tremblay, Jay M. J. R. Carr, Vitalie Faoro and Philip N. Ainslie, revised and edited the review. All authors approved the final version of the review and agree to be accountable for all aspects of the work in ensuring that questions related to the accuracy or integrity of any part of the work are appropriately investigated and resolved. All persons designated as authors qualify for authorship, and all those who qualify for authorship are listed.

## CONFLICT OF INTEREST

None declared.
